# Concordant high-grade glioma in monozygotic twins with germline variants in ATM, FANCC, and FANCM: a case report on combined DNA repair deficiency

**DOI:** 10.1093/noajnl/vdag172

**Published:** 2026-07-02

**Authors:** Paolo Aretini, Francesca Lessi, Francesca Di Lorenzo, Aldo Pastore, Mariangela Morelli, Orazio Santo Santonocito, Sara Franceschi, Mario Bianco, Chiara Maria Mazzanti

**Affiliations:** Fondazione Pisana per la Scienza (P.L., F.L., F.D.L., A.P., M.M., S.F., C.M.M.); Fondazione Pisana per la Scienza (P.L., F.L., F.D.L., A.P., M.M., S.F., C.M.M.); Fondazione Pisana per la Scienza (P.L., F.L., F.D.L., A.P., M.M., S.F., C.M.M.); Fondazione Pisana per la Scienza (P.L., F.L., F.D.L., A.P., M.M., S.F., C.M.M.); Fondazione Pisana per la Scienza (P.L., F.L., F.D.L., A.P., M.M., S.F., C.M.M.); Neurosurgical Department of Spedali Riuniti di Livorno, Livorno, Italy (O.S.S.); Fondazione Pisana per la Scienza (P.L., F.L., F.D.L., A.P., M.M., S.F., C.M.M.); Distretto Socio Sanitario 1, ASL BAT, Margherita di Savoia (M.B.); Fondazione Pisana per la Scienza (P.L., F.L., F.D.L., A.P., M.M., S.F., C.M.M.)

Glioblastoma (GBM) remains a fatal disease of predominantly sporadic origin.^1^^,^^2^ However, rare familial aggregation informs genetic predisposition.^3-5^ Monozygotic twins are a unique model to dissect germline susceptibility versus somatic evolution. Established syndromes explain some familial cases,^6^^,^^7^ but the impact of combined polygenic DNA repair defects remains understudied.^8-10^ FANCM is increasingly recognized not only as a core Fanconi Anemia (FA) gene but as a distinct cancer susceptibility factor, with variants driving early-onset malignancy without the full FA phenotype.^11^ Here, we report an exceptional case of concordant high-grade glioma in monozygotic twins diagnosed at ages 17 and 24. Whole exome sequencing identified a novel germline triad of variants in ATM, FANCC, and FANCM (Figure 1), which we propose act together to produce a “mild Fanconi-like” genomic instability phenotype, with implications for family counseling and targeted therapy.^12^

**Figure 1.**
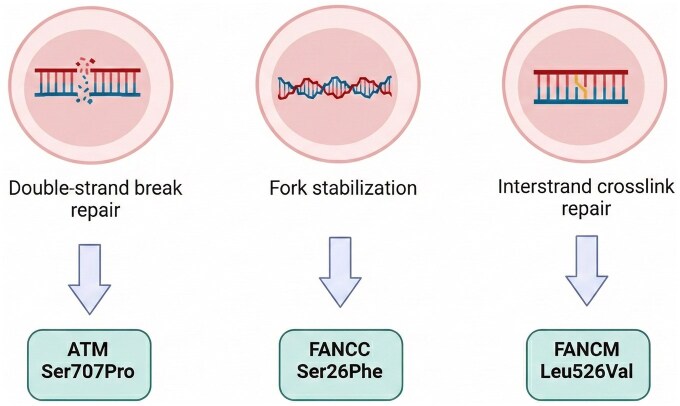
Schematic representation of the germline variant triad identified in monozygotic twins with concordant high-grade glioma. The variants involve ATM (Ser707Pro), FANCC (Ser26Phe), and FANCM (Leu526Val), each contributing to a distinct but functionally convergent aspect of the DNA damage response (DDR) and Fanconi anemia (FA) pathway. The combined impairment likely results in reduced double-strand break signaling, replication fork stability, and cross-link repair, producing a “mild Fanconi-like” phenotype predisposing to tumor development.

## Methods

### Case Reports

Case 1: Twin 1 presented at age 17 with progressive headaches and a right parieto-occipital intra-axial lesion. He underwent surgical resection; initial histology indicated pilocytic astrocytoma. Despite adjuvant radiotherapy and temozolomide, the tumor recurred, and the patient died 15 months after diagnosis.

Case 2: Twin 2 presented 7 years later, at age 24, with an inoperable corpus callosum lesion consistent with high-grade glioma on MR spectroscopy. He received supportive therapy and died four months after diagnosis.

### Family Context and Ethics

**Figure 2.**
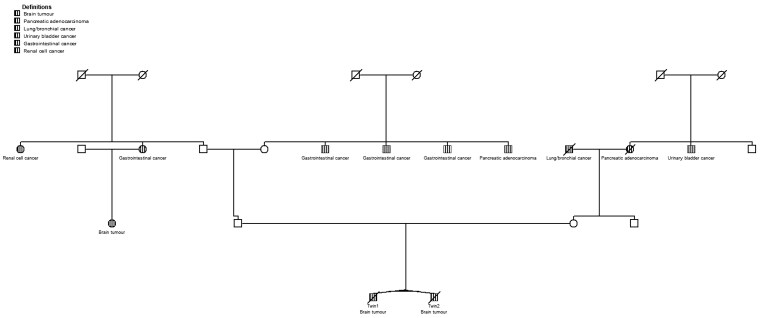
Three-generation pedigree of the proband family, reconstructed by Dr. Mario Bianco (maternal uncle and coauthor) from family records and clinical history. Squares indicate males; circles indicate females; diagonal lines indicate deceased individuals. Filled symbols indicate confirmed cancer diagnoses as indicated in the legend. Twin 1 and Twin 2 are indicated at the bottom of the pedigree (brain tumor). The paternal lineage shows gastrointestinal cancer (paternal grandfather), renal cell cancer (paternal uncle), and brain tumor in a second-degree paternal relative. The maternal lineage shows a heavy cancer burden including gastrointestinal cancer (3 maternal uncles), pancreatic adenocarcinoma (maternal grandmother and 1 maternal uncle), lung/bronchial cancer (one maternal uncle), and urinary bladder cancer (one maternal uncle). No molecular testing had been performed on any affected relative prior to this study. The bilateral accumulation of gastrointestinal, pancreatic, pulmonary, and brain malignancies across both lineages is consistent with an underlying hereditary DNA damage response deficiency.

Coauthor Dr. Mario Bianco is the maternal uncle of the twins and the neurosurgeon who performed the craniotomy on Twin 1. A 3-generation pedigree reconstructed by Dr. Bianco (Figure 2) revealed a substantial bilateral cancer burden: paternal side (gastrointestinal cancer, renal cell cancer, brain tumor in a second-degree relative) and maternal side (three gastrointestinal cancers, 2 pancreatic adenocarcinomas, 1 lung cancer, 1 bladder cancer). Written informed consent for scientific research use of biological samples and clinical data was obtained from both parents prior to analysis, in accordance with Legge 219/2017, D.Lgs. 101/2018/GDPR, and the Declaration of Helsinki. This study was not subject to formal approval by an institutional review board (IRB) or ethics committee, as the Fondazione Pisana per la Scienza is a private nonprofit research institution and the study was conducted as a retrospective research investigation on residual archival material with full parental consent. All procedures were conducted in accordance with the applicable Italian regulatory framework and the principles of the Declaration of Helsinki.

### Genomic Analysis

Peripheral blood DNA (parents and Twin 2) and archival FFPE tumor tissue (Twin 1) underwent whole exome sequencing (WES) using the Illumina DNA Prep with Exome 2.5 Enrichment kit on the Illumina NextSeq 2000 platform. Reads were aligned to GRCh38; germline variants were called with DRAGEN v4.2.7 and somatic variants with GATK Mutect2 v4.4.0.0 (tumor-normal mode, Twin 2 as matched normal). Monozygosity was confirmed by KING analysis (MZ = 1; concordance = 97.9%).^13^ Per-sample WES coverage metrics and KING results are reported in Supplementary Table S1. CNV analysis used CNVkit. Variants were annotated with GATK VariantAnnotator and OpenCravat and classified per ACMG/AMP guidelines. Full methodological details are provided in Supplementary Methods.

## Results

WES identified 3 heterozygous germline variants in DNA repair genes in both twins, absent in either parent, all classified as Variants of Uncertain Significance (VUS) under ACMG/AMP criteria. ATM p. Ser707Pro (c.2119T > C, NM_000051.4; gnomAD AF 7.74 × 10^−^³; CADD Phred 14.2; DANN Phred 11.05; not in ClinVar) was inherited from the father and affects the FAT domain involved in kinase activation. FANCC p. Ser26Phe (c.77C > T, NM_000136.3; gnomAD AF 5.02 × 10^−^³; CADD Phred 24.7; DANN Phred 25.10; ClinVar: conflicting) was inherited from the mother and affects an N-terminal residue critical for FA core complex assembly. FANCM p. Leu526Val (c.1576C > G, NM_020937.4; gnomAD AF 1.44 × 10^−^³; CADD Phred 13.9; DANN Phred 12.32; ClinVar: conflicting) was inherited from the father and affects the ATPase domain involved in replication fork remodeling. Full annotation is provided in Table 1.

**Table 1.** Germline variant annotation summary and somatic second hit in ATM

Gene	HGVS (protein)	HGVS (cDNA/transcript)	DNA repair pathway	gnomAD AF	CADD Phred	DANN Phred	ClinVar	ACMG	Inheritance	**VAF Twin 1** ^a^	**VAF Twin 2** ^b^
ATM	p.Ser707Pro	c.2119T > C (NM_000051.4)	DSB signaling/ATM kinase	7.74 × 10−³	14.2	11.05	Not in ClinVar	VUS	Father	0.39	0.54
FANCC	p.Ser26Phe	c.77C > T (NM_000136.3)	FA core complex/ICL repair	5.02 × 10−³	24.7	25.10	Conflicting	VUS	Mother	0.50	0.37
FANCM	p.Leu526Val	c.1576C > G (NM_020937.4)	FA core complex/fork remodeling	1.44 × 10−³	13.9	12.32	Conflicting	VUS	Father	0.50	0.57
**Somatic second hit (Twin 1 tumor only)**											
ATM	p.Arg1875Ter	c.5623C > T (NM_000051.4)	DSB signaling/ATM kinase	0.00 in germline	36	—	Pathogenic	Class 5	Somatic	0.024	n/a

Variant allele frequencies (VAF) are reported for Twin 1 (FFPE tumor) and Twin 2 (peripheral blood).

a Twin 1 VAF measured from FFPE tumor tissue; expected VAF for germline heterozygous variants is ∼0.5 but may be reduced due to FFPE-related artifacts, tumor purity, and allelic imbalance.

b Twin 2 VAF measured from peripheral blood (germline). Somatic second hit absent in Twin 2 germline (n/a). AF, allele frequency; ACMG, American College of Medical Genetics and Genomics; CADD, Combined Annotation Dependent Depletion (Phred-scaled); DANN, Deleterious Annotation of genetic variants using Neural Networks (Phred-scaled); DSB, double-strand break; FA, Fanconi anemia; ICL, interstrand cross-link; VAF, variant allele frequency; VUS, variant of uncertain significance. All germline variants classified per ACMG/AMP 2015 guidelines.

This germline triad was exclusive to the twins, establishing a unique genetic background that likely contributed to their concordant high-grade gliomas (Table 1).

To further elucidate the tumorigenesis mechanism, we performed somatic variant calling on the FFPE tumor tissue of Twin 1 using GATK Mutect2 v4.4.0.0 in tumor-normal mode (see Methods). CNV analysis identified statistically significant focal amplification of EGFR (*P* = .004) and PDGFRA (*P* = .0007), consistent with IDH-wildtype high-grade glioma. No IDH1 (codon 132) or IDH2 (codon 172) mutations were identified. Canonical TERT promoter mutations (C228T/C250T) were absent; however, their absence does not exclude IDH-wildtype GBM, as approximately 20%-30% of such tumors lack TERT promoter mutations, particularly in PDGFRA-amplified subtypes.^14^^,^^15^ Chromosomal-level copy-number assessment for +7/−10 was not possible from WES-based CNVkit data, which has limited resolution for whole-chromosome aneuploidies; this limitation is acknowledged. The somatic driver profile included pathogenic TP53 mutations (p.Arg273His and p. Arg175His) and a frameshift truncation in MDM2 (p.Leu346PhefsTer52). A somatic coding variant in TERT (p.Arg240His, c.719G > A) was identified as a variant of uncertain significance in the reverse transcriptase domain, distinct from canonical promoter hotspot mutations, and is reported accordingly. Critically, a somatic nonsense mutation in ATM was identified: c.5623C > T, p. Arg1875Ter (DANN Phred = 36; ClinVar: Pathogenic; VAF in tumor = 0.024; allele frequency = 0.00 in matched germline). The co-occurrence of this somatic truncating mutation with the germline heterozygous missense variant p. Ser707Pro (VAF in Twin 1 FFPE = 0.39; VAF in Twin 2 blood = 0.54; inherited from the father) constitutes classical Knudson 2-hit biallelic inactivation of ATM in the tumor, demonstrating directly that ATM functions as a tumor suppressor in this case. No somatic second hits were identified at the FANCC or FANCM loci (germline VAF: FANCC Twin 1 = 0.50, Twin 2 = 0.37; FANCM Twin 1 = 0.50, Twin 2 = 0.57; all VAF reported in Table 1). Tumor mutational burden (TMB), calculated from 1,259 coding somatic PASS variants over 52 Mb of exome target, was 24.2 mutations/Mb. This elevated TMB is mechanistically consistent with complete ATM loss, which abolishes double-strand break checkpoint signaling and has been associated with elevated somatic mutation rates in multiple tumor types. A somatic MSH6 missense variant (p.Arg1242Cys; CADD Phred = 32; VUS) was identified and is reported for completeness; however, a single heterozygous VUS without biallelic loss is insufficient to classify the tumor as MMR-deficient. Although both twins are deceased, tumor tissue from the second twin was not available for molecular comparison; somatic evolution in the second twin therefore cannot be directly assessed.

## Discussion

This concordant case of high-grade glioma in monozygotic twins illustrates how a polygenic DNA repair deficiency can drive aggressive brain tumors. The germline triad in ATM, FANCC, and FANCM converges on a common functional outcome: impaired genomic stability. ATM governs double-strand break signaling; FANCC and FANCM contribute to FA core complex function and replication fork remodeling, respectively.^9^ Their co-occurrence in the twins, absent in either parent, likely produced cumulative DDR impairment consistent with a polygenic predisposition model. The ATM -hit biallelic inactivation confirmed in Twin 1’s tumor provides direct mechanistic validation.

The ATM 2-hit inactivation is the dominant somatic driver identified in this case, consistent with the established role of complete ATM loss in promoting genomic instability and elevated tumor mutational burden. In addition to the ATM second hit, somatic analysis identified MDM2, and a somatic coding VUS in TERT (p.Arg240His; distinct from canonical promoter hotspot mutations) in the primary tumor. TP53 biallelic mutations and MDM2 frameshift truncation are consistent with high-grade glial neoplasm and likely represent precipitating events for malignant transformation.^16^ The absence of tumor DNA in the second twin makes it impossible to determine whether he had similar somatic drivers in his presumed high-grade glioma. However, given their similar clinical courses, this possibility is highly plausible. The stepwise model, in which a germline triad confers susceptibility and acquired somatic drivers complete the transformation, offers a coherent framework for understanding the disease in these twins. This finding is consistent with previous research. Central nervous system (CNS) tumors have been reported in cases of Fanconi anemia, particularly in association with mutations in the BRCA2/FANCD1 gene.^17^ The existing literature indicates that germline variants of the ATM and FANCM genes have been identified in cases of pediatric and early-onset gliomas. Mateos et al specifically reported germline DDR pathway alterations in a pediatric diffuse midline glioma cohort.^12^ More recently, large-scale analyses of adult GBM patients have confirmed that pathogenic germline variants in DDR genes–including ATM, BRCA1/2, and Fanconi anemia pathway genes—are present in 5%-10% of unselected cases and up to 23% of patients with a personal or family history of cancer.^18^^,^^19^ The present findings contribute to this body of literature by demonstrating that polygenic combinations of DDR variants can induce a “Fanconi-like” state of genomic instability beyond classical syndromes.

From a clinical perspective, these observations underscore the importance of germline testing for early-onset or familial gliomas. ATM heterozygosity has been shown to result in radiosensitivity, indicating the necessity for ­personalized radiotherapy approaches. At the same time, deficiencies in the Fanconi anemia (FA) or homologous recombination (HR) pathways could lead to targeted treatments, such as poly(ADP-ribose) polymerase (PARP) or ataxia-telangiectasia (ATR) inhibitors. These treatments have been shown to be effective in similar molecular environments.^12^^,^^20^ These results clearly demonstrate the importance of genetic counseling for family members, as they may carry variants that increase cancer risk.^21^

In conclusion, the convergence of germline DNA repair variants (ATM, FANCC, and FANCM) and somatic oncogenic drivers (TP53, MDM2) likely explains the development of concordant high-grade glioma in these monozygotic twins. A critical finding of this study is the demonstration of classical Knudson 2-hit biallelic inactivation of ATM in the tumor of Twin 1 (germline p. Ser707Pro combined with somatic nonsense p. Arg1875Ter), confirming ATM as a tumor suppressor in this context and providing direct molecular evidence linking the germline background to tumor development. The elevated tumor mutational burden (TMB = 24.2 mut/Mb) is mechanistically consistent with complete ATM loss. FANCC and FANCM heterozygosity is proposed to contribute a permissive background of genomic instability, consistent with a polygenic model of DNA repair deficiency. This case contributes to the emerging literature on oligogenic and polygenic cancer predisposition, shifting the focus toward combined DDR variant models in early-onset glioma. It underscores the importance of germline WES in young patients with glioma to identify DNA repair defects that may inform family counseling and guide targeted therapeutic strategies.

### Limitations

This study has several limitations that should be acknowledged. First, DNA methylation profiling (Illumina EPIC 850K array), which would provide the most robust molecular classification of Twin 1’s tumor under the WHO 2021 CNS classification framework, was not performed. This analysis was not available at the time of diagnosis and is not within the technical capabilities of our laboratory. We note, however, that the WES-based molecular profile independently satisfies criteria for IDH-wildtype high-grade glioma under WHO 2021: IDH-wildtype status confirmed, EGFR amplification (*P* = .004), PDGFRA amplification (*P* = .0007), and biallelic TP53 mutation. This molecular evidence is considered sufficient for classification in the absence of methylation data, as endorsed by cIMPACT-NOW update 3. Second, external neuropathological review of the original histological slides from Twin 1 could not be performed, as the archival material from the referring hospital was no longer available for retrieval. The molecular characterization provided by WES therefore constitutes the primary basis for the revised diagnosis, superseding the initial histopathological classification of pilocytic astrocytoma, which is considered inconsistent with the somatic driver profile and clinical course.

## Supplementary Material

Supplementary material is available *online at Neuro-Oncology Advances* (https://academic.oup.com/noa).

## Supplementary Material

vdag172_Supplementary_Data

## Data Availability

The data that support the findings of this study are available from the corresponding author upon reasonable request. Raw sequencing data are not publicly deposited due to privacy restrictions related to the nature of the case (familial cancer genetics); de-identified processed data may be shared upon reasonable request and subject to applicable data protection regulations.

## Dedication

To my beloved nephews: monozygotic twins genetically bound from their beginning to their untimely end. A tragic destiny that has nonetheless revealed itself, through research, as a hope for scientific progress in understanding and treating glioblastoma, the most devastating of brain tumors. This article is the result of a collaboration between the clinical neurosurgeon and researcher Chiara Mazzanti, stemming from a chance meeting at a congress dinner in 2024. On that occasion, we discussed the potential genetic aspects of my nephews’ case. I view this serendipity as almost mystical, a twist of destiny: I had preserved my nephew’s blood sample for years, eventually forgetting about it, until this meeting paved the way for the current findings. Mario Bianco, uncle and author.

## References

[1] Ostrom QT , Price M, Neff C, et al CBTRUS statistical report: primary brain and other central nervous system tumors diagnosed in the United States in 2015–2019. Neuro Oncol. 2022;24:v1-v95. 10.1093/neuonc/noac202 36196752 PMC9533228

[2] Shah S. Novel therapies in glioblastoma treatment: review of glioblastoma; current treatment options; and novel oncolytic viral therapies. *Med Sci*. 2023;12: 1. 10.3390/medsci12010001 PMC10801585 38249077

[3] Choi D-J , Armstrong G, Lozzi B, et al; Genomics England Research Consortium. The genomic landscape of familial glioma. Sci Adv. 2023;9:eade2675. 10.1126/sciadv.ade2675 37115922 PMC10146888

[4] Scheurer ME , Etzel CJ, Liu M, et al; GLIOGENE Consortium. Familial aggregation of glioma: a pooled analysis. Am J Epidemiol. 2010;172:1099-1107. 10.1093/aje/kwq261 20858744 PMC3025634

[5] Purow B , Schiff D. Advances in the genetics of glioblastoma: are we reaching critical mass? Nat Rev Neurol. 2009;5:419-426. 10.1038/nrneurol.2009.96 19597514 PMC3387541

[6] Dunbar EM , Eppolito A, Henson JW. Genetic counseling and tumor predisposition in neuro-oncology practice. Neurooncol Pract. 2016;3:17-28. 10.1093/nop/npv051 31579518 PMC6760343

[7] Louis DN , Perry A, Wesseling P, et al The 2021 WHO classification of tumors of the central nervous system: a summary. Neuro Oncol. 2021;23:1231-1251. 10.1093/neuonc/noab106 34185076 PMC8328013

[8] Jovanović A , Tošić N, Marjanović I, et al Germline variants in cancer predisposition genes in pediatric patients with central nervous system tumors. Int J Mol Sci. 2023;24:17387. 10.3390/ijms242417387 PMC10744041 38139220

[9] Ceccaldi R , Sarangi P, D’Andrea AD. The Fanconi anaemia pathway: new players and new functions. Nat Rev Mol Cell Biol. 2016;17:337-349. 10.1038/nrm.2016.48 27145721

[10] Del Valle J , Rofes P, Moreno-Cabrera JM, et al Exploring the role of mutations in Fanconi anemia genes in hereditary cancer patients. Cancers (Basel). 2020;12:829. 10.3390/cancers12040829 32235514 PMC7226125

[11] Fang C , Zhu Z, Cao J, et al Comprehensive review on Fanconi anemia: insights into DNA interstrand cross-links, repair pathways, and associated tumors. Orphanet J Rare Dis. 2025;20:389. 10.1186/s13023-025-03896-w 40739565 PMC12312369

[12] Mateos MK , Ajuyah P, Fuentes-Bolanos N, et al Germline analysis of an international cohort of pediatric diffuse midline glioma patients. Neuro Oncol. 2025;27:1849-1863. 10.1093/neuonc/noaf061 40072012 PMC12417819

[13] Manichaikul A , Mychaleckyj JC, Rich SS, Daly K, Sale M, Chen W-M. Robust relationship inference in genome-wide association studies. Bioinformatics. 2010;26: 2867-2873. 10.1093/bioinformatics/btq559 20926424 PMC3025716

[14] Diplas BH , He X, Brosnan-Cashman JA, et al The genomic landscape of TERT promoter wildtype-IDH wildtype glioblastoma. Nat Commun. 2018;9:2087. 10.1038/s41467-018-04448-6 29802247 PMC5970234

[15] Fujimoto K , Arita H, Satomi K, et al Tert promoter mutation status is necessary and sufficient to diagnose IDH-wildtype diffuse astrocytic glioma with molecular features of glioblastoma. *Acta Neuropathol*. 2021;142: 323-338. 10.1007/s00401-021-02337-9 34148105

[16] Reifenberger G , Wirsching H-G, Knobbe-Thomsen CB, et al Advances in the molecular genetics of gliomas–implications for classification and therapy. Nat Rev Clin Oncol. 2017;14:434-452. 10.1038/nrclinonc.2016.204 28031556

[17] McReynolds LJ , Biswas K, Giri N, et al Genotype-cancer association in patients with Fanconi anemia due to pathogenic variants in FANCD1 (BRCA2) or FANCN (PALB2). Cancer Genet. 2021;258-259:101-109. 10.1016/j.cancergen.2021.10.001 34687993 PMC8628873

[18] Van Opijnen MP , Van Valkengoed DR, De Ligt J, et al Whole genome sequencing-based analysis of genetic predisposition to adult glioblastoma. *NPJ Genom Med*. 2025;10: 70. 10.1038/s41525-025-00526-z 41168197 PMC12575641

[19] Brand F , Rose LS, Akbarzadeh AH, et al Germline variants in ATM, BRCA2, other cancer predisposition and novel candidate genes are implicated in glioma risk in adult glioma patients with a familial or personal history of tumors. *Acta Neuropathol*. 2026;151: 6. 10.1007/s00401-025-02972-6 41546701 PMC12812103

[20] Mateo J , Lord CJ, Serra V, et al A decade of clinical development of PARP inhibitors in perspective. Ann Oncol. 2019;30:1437-1447. 10.1093/annonc/mdz192 31218365 PMC6771225

[21] Riley BD , Culver JO, Skrzynia C, et al Essential elements of genetic cancer risk assessment, counseling, and testing: updated recommendations of the National Society of Genetic Counselors. J Genet Couns. 2012;21:151-161. 10.1007/s10897-011-9462-x 22134580

