# Supplementary material for: Concordant high-grade glioma in monozygotic twins with germline variants in ATM, FANCC, and FANCM: a case report on combined DNA repair deficiency
Source: Neurooncol Adv. 2026 Jul 2;8(1):vdag172. doi: 10.1093/noajnl/vdag172 (PMC13395281; doi:10.1093/noajnl/vdag172)
Supplement: vdag172_Supplementary_Data [file vdag172_supplementary_data.zip › Supplementary_Table_S1.docx]

**Supplementary Table S1**

**Whole exome sequencing quality metrics and KING monozygosity analysis.**

**Panel A. Per-sample WES coverage metrics**

| **Sample** | **Sample Type** | **Mean Region Coverage (×)** | **Uniformity of Coverage (%)** | **Role in Analysis** |
| --- | --- | --- | --- | --- |
| Mother | Germline blood | 103.63 | 97.93 | Germline variant calling; segregation analysis |
| Father | Germline blood | 131.97 | 97.79 | Germline variant calling; segregation analysis |
| Twin 2 | Germline blood | 47.33 | 95.74 | Matched normal for Mutect2 somatic calling; CNVkit reference; KING monozygosity analysis |
| Twin 1 | FFPE tumor | 231.7 | 99.06 | Somatic variant calling (Mutect2 tumor-normal); CNV analysis (CNVkit) |
| *Coverage and uniformity metrics computed by DRAGEN v4.2.7 (Illumina) from alignment to GRCh38 reference genome. Uniformity of coverage: percentage of targeted bases with coverage ≥ 0.2× mean. All samples sequenced on the Illumina NextSeq 2000 platform using the Illumina DNA Prep with Exome 2.5 Enrichment kit. Minimum accepted threshold for germline WES: ≥30×; all samples exceed this threshold. FFPE, formalin-fixed paraffin-embedded.* | | | | |

**Panel B. KING genome-wide monozygosity analysis**

| **Sample Pair** | **KING Relationship Inference** | **Kinship Coefficient** | **Genome-wide Genotype Concordance** | **Notes** |
| --- | --- | --- | --- | --- |
| Twin 1 (FFPE) vs Twin 2 (blood) | Monozygotic Twins (MZ = 1) | 0.500 (MZ) | 0.979 (97.9%) | Residual discordance (2.1%) attributable to FFPE-related sequencing artefacts; expected for MZ comparisons involving FFPE-derived DNA |
| *Kinship analysis performed using KING v2.3 (Manichaikul et al., Bioinformatics 2010; 26:2867–2873). KING classifies monozygotic twin pairs based on kinship coefficient ≥ 0.354 and IBS0 rate ≈ 0. Genome-wide genotype concordance computed from germline SNP calls (Twin 2 blood WES vs Twin 1 FFPE tumor WES). The observed concordance of 97.9% is fully consistent with MZ status; the 2.1% discordance reflects FFPE-related sequencing artefacts (deamination, fragmentation) rather than true genetic differences between the twins, as confirmed by KING MZ inference. This result retrospectively validates the use of Twin 2 germline WES as matched normal for somatic variant calling in Twin 1.* | | | | |
