# Supplementary material for: Concordant high-grade glioma in monozygotic twins with germline variants in ATM, FANCC, and FANCM: a case report on combined DNA repair deficiency
Source: Neurooncol Adv. 2026 Jul 2;8(1):vdag172. doi: 10.1093/noajnl/vdag172 (PMC13395281; doi:10.1093/noajnl/vdag172)
Supplement: vdag172_Supplementary_Data [file vdag172_supplementary_data.zip › Supplementary_Methods.docx]

**Supplementary Methods**

*Concordant High-Grade Glioma in Monozygotic Twins with Germline Variants in ATM, FANCC, and FANCM: A Case Report on Combined DNA Repair Deficiency*

**1. Case Reports**

**Case 1**

Twin 1 presented at age 17 with progressive headaches. A CT scan revealed a right parieto-occipital intra-axial lesion. The patient underwent urgent surgical resection shortly after presentation. Initial histological analysis indicated a diagnosis of pilocytic astrocytoma. Despite receiving adjuvant radiotherapy and temozolomide, the tumor recurred in the corpus callosum. The patient's condition deteriorated rapidly, resulting in death 15 months after the initial diagnosis.

**Case 2**

Twin 2 presented with worsening headaches seven years later, at the age of 24. Imaging revealed a substantial lesion in the corpus callosum and MR spectroscopy indicated a high-grade glioma. The tumor was deemed inoperable. The patient received corticosteroids and mannitol for three months. A period of clinical stability followed. However, his condition deteriorated, resulting in death four months after diagnosis.

**2. Family Context and Ethics**

Co-author Dr. Mario Bianco is the maternal uncle of the twins and the neurosurgeon who performed the craniotomy on Twin 1. Following the deaths of both nephews from concordant brain tumors, Dr. Bianco preserved the biological material and facilitated the research collaboration described in the Dedication. The genomic analysis was conducted at the Fondazione Pisana per la Scienza (FPS) as a research investigation.

A three-generation pedigree was reconstructed by Dr. Bianco from family records and clinical history (Figure 2). The paternal lineage showed a history of gastrointestinal cancer (paternal grandfather) and renal cell cancer (paternal uncle), with one second-degree relative on the paternal side affected by brain tumor. The maternal lineage revealed a substantially heavier cancer burden: three maternal uncles affected by gastrointestinal cancer, one maternal uncle with pancreatic adenocarcinoma, one maternal uncle with lung/bronchial cancer, the maternal grandmother with pancreatic adenocarcinoma, and one maternal uncle with urinary bladder cancer. No molecular testing had previously been performed on any affected relative. The bilateral accumulation of gastrointestinal, pancreatic, lung, and brain cancers across both lineages is consistent with an underlying hereditary DNA repair deficiency, providing important familial context for the germline triad identified in the twins.

Written informed consent for the use of biological samples and clinical data for scientific research purposes was obtained from both parents prior to initiation of the genomic analysis, in accordance with the applicable Italian regulatory framework (Legge 219/2017; D.Lgs. 101/2018 implementing GDPR) and the principles of the Declaration of Helsinki. Both parents were informed of the germline findings identified in this study and expressed their autonomous desire to understand the genetic cause of the disease affecting their sons.

**3. DNA Extraction**

Total genomic DNA was extracted from whole blood samples (200 μL) using the automated EZ1&2 DNA Blood 200 μL Kit (Qiagen, Hilden, Germany) on the EZ2 Connect workstation, following the manufacturer’s instructions. This magnetic-bead-based technology ensured automated purification in a final elution volume of 100 μL. The concentration of the isolated DNA was subsequently determined using the Qubit dsDNA BR (Broad Range) Assay Kit (Thermo Fisher Scientific, Waltham, MA, USA) on a Qubit Fluorometer.

**4. Whole Exome Sequencing**

Peripheral blood DNA from both parents and Twin 2, and archival formalin-fixed, paraffin-embedded (FFPE) tumor tissue from Twin 1, underwent whole exome sequencing (WES) using the Illumina DNA Prep with Exome 2.5 Enrichment kit on the Illumina NextSeq 2000 platform. Reads were aligned to GRCh38. Per-sample coverage metrics are reported in Supplementary Table S1: mother 103.63× (uniformity 97.93%), father 131.97× (97.79%), Twin 2 blood 47.33× (95.74%), Twin 1 FFPE tumor 231.7× (99.06%).

**5. Germline Variant Calling and Annotation**

Germline variant calling was performed using the DRAGEN v4.2.7 germline pipeline (Illumina). PASS filter was applied throughout. Variants were annotated with GATK VariantAnnotator and OpenCravat. Germline variant deleteriousness was assessed using DANN (Deleterious Annotation of genetic variants using Neural Networks; Phred-scaled scores) and CADD (Combined Annotation Dependent Depletion; Phred-scaled scores). Population allele frequencies were derived from gnomAD v3.1.2. All variants were classified according to the ACMG/AMP 2015 guidelines. Segregation analysis was performed on both parents.

**6. Monozygosity Analysis**

Monozygosity was confirmed by genome-wide kinship analysis using KING v2.3 (Manichaikul et al., Bioinformatics 2010; 26:2867–2873). KING classifies monozygotic twin pairs based on kinship coefficient ≥ 0.354 and IBS0 rate ≈ 0. The analysis yielded MZ = 1 by genomic inference, with genome-wide genotype concordance of 97.9% between Twin 2 (blood) and Twin 1 (FFPE tumor). The 2.1% residual discordance is attributable to FFPE-related sequencing artefacts (deamination, fragmentation) rather than true genetic differences, as confirmed by KING MZ inference. Full KING results are reported in Supplementary Table S1.

**7. Somatic Variant Calling**

Somatic variant calling was performed on the FFPE tumor tissue of Twin 1 using GATK Mutect2 v4.4.0.0 in tumor-normal mode. Twin 2 germline WES was used as the matched normal. Additional Mutect2 inputs included a panel of normals (1000 Genomes PoN: 1000g_pon.hg38.vcf) and a germline resource (af-only-gnomad.hg38.vcf). Post-processing included: LearnReadOrientationModel to correct FFPE deamination bias, GetPileupSummaries and CalculateContamination for contamination estimation, and FilterMutectCalls with OPTIMAL_F_SCORE thresholding (FDR = 0.05).

Tumor mutational burden (TMB) was calculated as the number of coding somatic PASS variants divided by the effective exome target size: 1,259 coding somatic PASS variants / 52 Mb = 24.2 mutations/Mb.

**8. Copy Number Variation Analysis**

Copy number variation (CNV) analysis was performed using CNVkit (v0.9.x) with Twin 2 germline WES as the reference sample. This approach enables WES-based focal CNV detection. Statistically significant focal amplifications were identified for EGFR (p = 0.004) and PDGFRA (p = 0.0007). Chromosomal-level copy-number assessment for +7/−10 was not possible from WES-based CNVkit data, which has limited resolution for whole-chromosome aneuploidies; this limitation is acknowledged in the manuscript.

*All analyses were performed on data aligned to GRCh38. All software versions and parameters are as described above. Raw and processed data are available from the corresponding author upon reasonable request.*
